# Validation of a guideline-based composite outcome assessment tool for asthma control

**DOI:** 10.1186/1465-9921-8-26

**Published:** 2007-03-21

**Authors:** Sally Spencer, Bhabita Mayer, Kate L Bendall, Eric D Bateman

**Affiliations:** 1 Brunel University, Uxbridge, UK; 2 GlaxoSmithKline Research & Development, Greenford, UK; 3 Giant Group PLC, London, UK; 4 University of Cape Town, Cape Town, South Africa

## Abstract

**Background:**

A global definition of asthma control does not currently exist. The purpose of this study was to validate two new guideline-based composite measures of asthma control, defined as totally controlled (TC) asthma and well controlled (WC) asthma.

**Methods:**

We used data from 3416 patients randomised and treated in the multi-centre Gaining Optimal Asthma controL (GOAL) study. The criteria comprising the asthma control measures were based on Global Initiative for Asthma/National Institutes of Health guidelines. This validation study examined the measurement properties of the asthma control measures using data from run-in, baseline, 12 and 52 weeks. Forced expiratory volume in 1 second (FEV_1_) and the Asthma Quality of Life Questionnaire (AQLQ) were used as the reference criteria in the validation analysis.

**Results:**

Both measures had good discriminative ability showing significant differences in FEV_1 _and AQLQ scores between control classification both cross-sectionally and longitudinally (p < 0.001). Overall both of the composite measures accounted for more of the variance in FEV_1 _after 52 weeks than the individual components of each asthma control measure. Both of the reference criteria were independently related to each asthma control measure (p < 0.0001). The measures also had good predictive validity showing significant differences in FEV_1 _and AQLQ scores at 52 weeks by control classification at 12 weeks (p < 0.0001).

**Conclusion:**

The guideline-based composite asthma control measures of WC asthma and TC asthma have good psychometric properties and are both valid functional indices of disease control in asthma.

## Background

The aim of asthma management, as endorsed by the publication of recent guidelines, is to achieve and maintain effective control of the disease [1-3]. The effectiveness of therapeutic interventions in asthma clinical trials is most commonly evaluated using individual endpoints, such as forced expiratory volume in 1 second (FEV_1_). However, the assessment of these individual endpoints may lead to an over-estimation of the level of asthma control achieved [4] and may not reflect the wider overall impact of the disease on the patient [5]. In addition, therapeutic benefit is frequently expressed in terms of the degree of change in individual endpoints, rather than the achievement of a concrete predefined clinical goal, such as the absence of key symptoms. While the former is a primary aim of research-led studies largely designed to evaluate the efficacy of new treatments, the latter is the primary clinical objective in the day-to-day management of asthma.

Composite outcome measures that incorporate a range of endpoints in a single definition allow a range of important disease characteristics to be taken into account. In chronic obstructive pulmonary disease this type of composite measure has been shown to have superior predictive properties, compared to the individual endpoints comprising the composite [6].

### Assessment of asthma control

Our aim was to validate two guideline-based measures of asthma control. These tools had 'yes' or 'no' decision-making properties because their primary function was as an index of clinical status for use in asthma management. The control criteria were based on definitions of asthma control in recently published guidelines, including Global Initiative for Asthma [3,7,8]. Totally controlled (TC) and well controlled (WC) asthma include seven endpoints, as detailed in Table 1. The evaluation of daytime symptoms and frequency of rescue β_2_-agonist medication are well-accepted measures for detecting deterioration of asthma [11], and impending exacerbations [12]. The presence of night-time awakening [13] or an emergency visit are important but different indicators of change in asthma status. Adverse events are included as a 'cost' of asthma control in the reference guidelines and were included in order to remain consistent with these (see Table 1). Morning peak expiratory flow (PEF) is considered to serve as an objective measure of asthma control supplementary to that of symptoms [9,10,14,15]. Any one of these criteria alone is considered insufficient in the definition of asthma control because although not independent of one another, each endpoint contributes unique information to the overall assessment [11,16-18].

**Table 1:** Definitions of well controlled (WC) and totally controlled (TC) asthma based on Global Initiative for Asthma (GINA)/National Institutes of Health (NIH) guideline aims of treatment [3, 8]

	**Goals of GINA/NIH**	**Totally controlled**	**Well controlled**
		**Each week all of:**	**Each week two or more of:**

**Daytime symptoms**	Minimal (ideally no)	None	≤ 2 days symptom score > 1
**Rescue β**_2_**-agonist use**	Minimal (ideally no)	None	Use on ≤ 2 days and ≤ 4 occasions per week
**Morning peak expiratory flow**	Near normal	≥ 80% predicted every day	≥ 80% predicted every day
			**All of:**
**Night-time awakening**	Minimal (ideally no)	None	None
**Exacerbations**	Minimal (infrequent)	None	None
**Emergency visits**	No	None	None
**Treatment-related adverse events**	Minimal	None enforcing change in asthma therapy	None enforcing change in asthma therapy

TC and WC asthma were defined as achievement of all of the specified criteria for that week. Asthma control was achieved if the patient recorded 7 out of 8 controlled weeks prior to each clinic visit. Baseline control was assessed over a 4-week period. Predicted peak expiratory flow (PEF) was calculated based on the ECSC standards for patients 18 years and older and on the Polgar standards for patients 12–17 years old.

Symptom score: 1 was defined as 'symptoms for one short period during the day'. Overall scale: 0 (none) – 5 (severe).

Exacerbations were defined as deterioration in asthma requiring treatment with an oral corticosteroid or an emergency department visit or hospitalisation.

The GOAL study specified two target levels of control (TC and WC) because, while suggesting that complete absence of symptoms of asthma was possible (TC), the GINA guidelines suggest that 'minimal' daytime symptoms and β_2_-agonist use are acceptable in 'controlled asthma' (WC). WC weeks were defined by achievement of all the specified criteria for that week. Asthma control was assessed over an 8-week period prior to each clinic visit. TC or WC asthma was achieved if the patient had at least 7 out of 8 weeks in that control state. Emergency visits, exacerbations or treatment-related adverse events during the 8-week period resulted in automatic failure of either TC or WC status for the whole period. TC asthma was defined as no symptoms or rescue medication use whereas WC asthma allowed a low level of symptoms and rescue medication use during the assessment period [19].

A set of pragmatic, clinical and psychometric criteria have been described as the minimum standards required in developing tools of this kind [20]. It is essential that the effectiveness of a new measure, such as a composite endpoint, be judged against widely accepted standards for the development of such tools. Therefore it is the responsibility of the developers to demonstrate that the tool has three essential properties. That it is reliable, i.e. that it can consistently yield the same results when administered on several occasions to the same stable patients and that it can discriminate between patients with differing levels of disease [21,22]. That it is valid, i.e. that it is measuring what it claims to measure. Lastly, that it has sufficient sensitivity, i.e. that it responds to changes in the underlying disease [22-24]. Rather than relying on a single established reference measure of disease activity against which to test the properties of the new instrument, we used multiple reference measures that together provide a profile of psychometric performance [24-27].

The aim of this study was to examine the reliability, validity and sensitivity of the two new guideline-based composite measures of control, TC and WC asthma, used in the Gaining Optimal Asthma controL (GOAL) study [19].

## Methods

### Study and population

**Figure 1:**
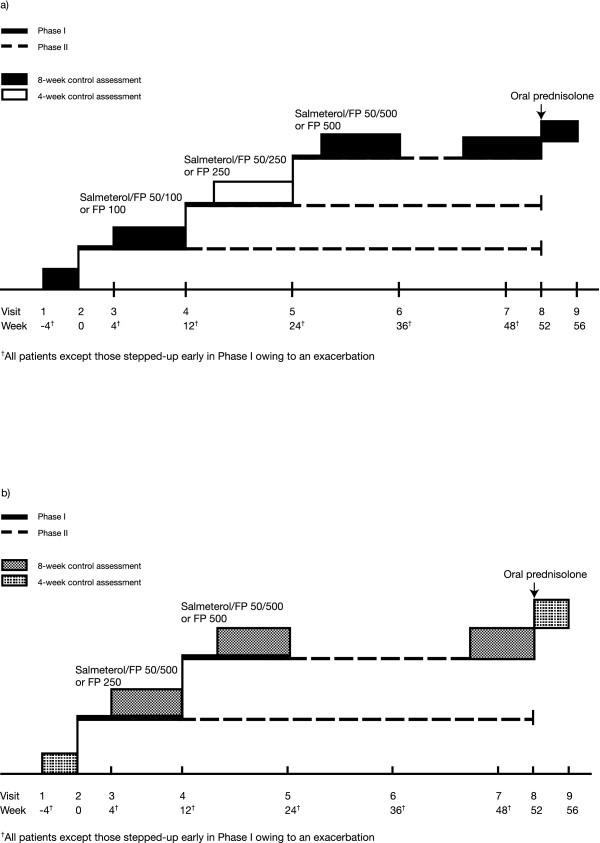
Study design for (a) Strata 1 and 2, and (b) Stratum 3. Following a 4-week run-in period, patients were randomised to receive either salmeterol/fluticasone propionate (SFC) or fluticasone propionate (FP) alone and stratified according to inhaled corticosteroid (ICS) use during the 6 months before screening: Stratum 1, no ICS; Stratum 2, 500 μg beclometasone dipropionate daily or equivalent; or Stratum 3, > 500–1000 μg beclometasone dipropionate daily or equivalent.

The GOAL study was a 1-year, randomised, stratified, double-blind, parallel-group trial comparing the efficacy of salmeterol/fluticasone propionate with fluticasone propionate alone in achieving two composite measures of asthma control. The study was conducted in 326 centres across 44 countries. Full details of the study are published elsewhere [19] and the study design is shown in Figure 1. During the 4-week run-in period patients who did not achieve at least two WC weeks were randomised to the study. During Phase I, the dose-escalation phase, treatment was 'stepped up' every 12 weeks until TC asthma was achieved or the highest dose of study drug reached. Patients entered Phase II either after achieving TC asthma or after 12 weeks on the maximum dose of study medication. During Phase II, patients remained on the dose at which they achieved TC asthma or the maximum dose of study medication until the end of the 1-year double-blind treatment period. Patients who failed to achieve TC asthma in Phase I were reassessed at the end of Phase II (Weeks 44–52).

Patients recruited to the study had at least a 6-month history of asthma and an improvement in FEV_1 _≥ 15% (and ≥ 200 ml) after inhalation of a short-acting β_2_-agonist. The mean age of patients in the study was 40.4 (range, 9–83), they had a mean baseline FEV_1 _of 74.47% of predicted (standard deviation, 18.55) and 42% were male. 5068 patients were screened and 3416 were randomised to the study and treated.

### Study protocol and outcomes

Asthma control was assessed over the 4-week run-in period and 8 weeks prior to each clinic visit at 12, 24, 36 and 52 weeks. For the purpose of validation we used data from the run-in period, baseline, 12 and 52 weeks. At the clinic visits, morning pre-dose FEV_1_, information regarding exacerbations, emergency visits and adverse events were recorded. In 197 centres in 16 countries health-related quality of life (HRQoL) was assessed using the Asthma Quality of Life Questionnaire (AQLQ) [28]. The AQLQ is a 32-item measure with established validity and reliability in asthma clinical trials [29-31]. It is scored from 0 (maximum impairment) to 7 (no impairment) and a within-subject change in score of ≥ 0.5 is considered the minimal clinically important difference [32,33].

### Validation analysis

Validation of composite endpoints is not straightforward when considering a measure that produces a 'state' rather than a 'score'. Traditional psychometric tests largely apply parametric statistics to continuous outcome variables. Given that our outcomes are not scores that can be defined as continuous variables, we have used the following methods to assess the validity of the measures of asthma control. Treatment was constant for all patients during Weeks 5–12 of Phase I, so the validation tests have focused on data from this period. The validation tests have been carried out on combined data from both treatment groups. In addition, since we are validating each of the composite measures of TC and WC asthma the validation tests have focused on comparisons between patients achieving the given control state and those who did not. For tests of sensitivity against the reference criteria we have used change from baseline data for the maximum possible period, i.e. to the end of the study at 52 weeks.

#### Construct validity

Traditionally, the construct validity of a new measure would be evaluated using a test such as Cronbach's Alpha to assess internal consistency, i.e. the degree to which all components contribute to the overall measure. In the absence of an overall score for the measure of asthma control, it was not possible to calculate the Alpha statistic. However, an assumption of composite endpoints in general is that all elements make a relatively independent contribution to that definition. We tested this assumption by examining the correlations amongst the seven criteria using the phi coefficient for dichotomous variables [26].

#### Reliability

Reliability would normally be tested using the intraclass correlation coefficient to establish within versus between occasion error variance. However, this was not possible during the study as a patients asthma control status was expected to fluctuate during the run-in period and to improve whilst on study medication. Therefore there was no predictable period of stability in which to test reliability at the group level.

#### Validity

In the absence of a gold standard against which to compare the composite measures of asthma control we used two reference criteria evaluated during the 1-year trial: percent predicted FEV_1 _as an objective index of airflow limitation; and the AQLQ as an established reference criterion for HRQoL that represents the impact of asthma on global health and well-being. Neither of these two measures formed part of the composite endpoints and were therefore appropriate as external reference criteria. However, the AQLQ was only evaluated in a sub-sample of the total population so preference is given to comparisons with percent predicted FEV_1 _where a larger proportion of the overall population provided information.

Validity of both the TC and WC definitions were evaluated cross-sectionally using an analysis of variance model to compare differences in the reference criteria values between patients who achieved the control status and those who did not. Longitudinal validity of both definitions was similarly tested by examining differences in change from baseline in the reference criterion between patients who achieved the control status and those who did not.

To evaluate the overall discriminative properties of the composite measures of asthma control we evaluated the discriminative properties of each composite endpoint (TC/WC) against the discriminative properties of the seven components of the composite endpoints. This was done through comparing the variation in percent predicted FEV_1 _attributed to the composite measures against the variation attributed to each component of the composite measures individually. The validity of the composite measures depends on the ability to discriminate between patients more effectively than the individual elements of the composite.

Finally, if the measure of asthma control summarises a range of aspects of disease impact, it should be related to each of our external reference criteria. A logistic regression model of control status with the reference criterion as factors was used to show how each reference criterion was related to control status, the amount of variation in the control status the two reference criterion accounted for was also assessed.

#### Predictive validity

To test the ability of the asthma control measures to predict future disease activity the Week 52 percent predicted FEV_1 _values were compared between patients who met the control criteria at Week 12 and those who did not. This was done using an analysis of variance model for each asthma control measure, and was repeated for the Week 52 AQLQ scores.

## Results

### Construct validity

Correlations between components of each control measure at Week 12 were moderate to weak (Table 2). β_2_-agonist use, night-time awakenings and daytime symptoms were, as expected, the most strongly correlated (r = 0.37 to 0.68). Results indicate that the individual criteria of the composite endpoint are relatively independent of each other. Very small correlations between emergency visits (EVs) and adverse events (AEs) were to be expected given the small number of each of these events (between Weeks 5 and 12 there were 91 EVs and 2 AEs). The relatively strong correlation between exacerbations and EVs was to be expected given that most of the emergency room visits were due to an exacerbation of patients' conditions.

**Table 2:** Correlation between control status criteria at Week 12

	β_2 _use	PEF	N-T awake	Exac.	EV	AE
**Well controlled**						
Symptoms	0.52	0.23	0.37	0.13	0.13	0.05
β_2 _use		0.28	0.46	0.13	0.11	0.03
PEF			0.24	0.10	0.08	0.04
N-T awake				0.12	0.09	0.04
Exac.					0.49	0.13
EV						0.18
**Totally controlled**						
Symptoms	0.68	0.25	0.38	0.09	0.08	0.01
β_2 _use		0.26	0.45	0.10	0.09	0.01
PEF			0.24	0.10	0.08	0.04
N-T awake				0.12	0.09	0.04
Exac.					0.49	0.13
EV						0.18

Symptoms = Daytime symptoms; β_2 _use = Use of rescue β_2_-agonist; PEF = Peak expiratory flow rate; N-T awake = Night time awakening; Exac. = exacerbations; EV = Emergency visits; AE = Treatment-related adverse events.

### Discriminative properties

**Figure 2:**
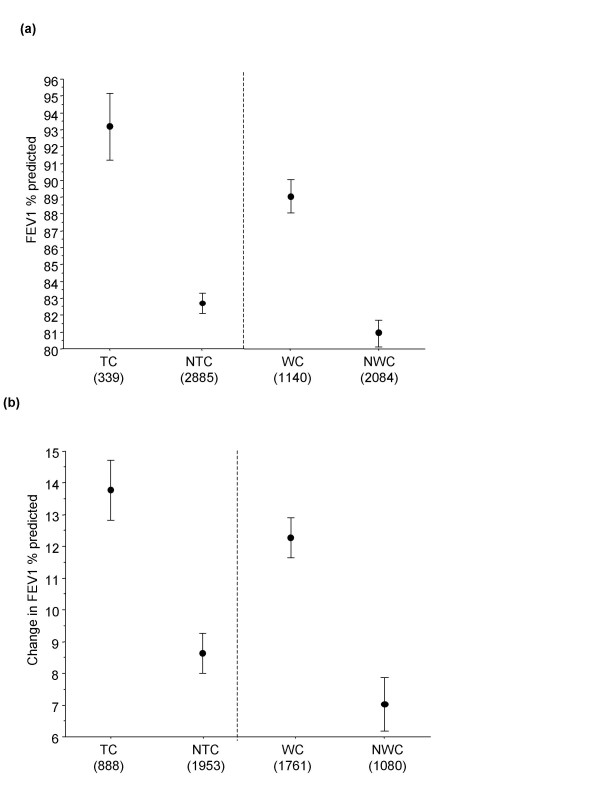
(a) Percent predicted forced expiratory volume in 1 second (FEV_1_) by control status at Week 12 (mean and 95% confidence interval) and (b) Change in percent predicted FEV_1 _from baseline to Week 52 by control status at Week 52 (mean and 95% confidence interval). Patients with TC asthma has significantly higher FEV_1 _% predicted compared to those with NTC asthma, and WC patients had significantly higher FEV_1 _% predicted compared to those NWC. *Key: *TC: Patients with totally controlled asthma. NTC: Patients with well controlled or not well controlled asthma. WC: Patients with either well controlled or totally controlled asthma. NWC: Patients with not well controlled asthma. Figures in brackets are number of patients per group.

The asthma control measures had good discriminative properties when compared with percent predicted FEV_1 _at Week 12 (Figure 2a) and change in percent predicted FEV_1 _from baseline to Week 52 (Figure 2b). Patients achieving TC asthma had higher percent predicted FEV_1 _values and showed greater improvements over 52 weeks compared to those with 'not' TC (NTC) asthma (p < 0.001). Similar results were seen for the WC definition. FEV_1 _improvements in patients with TC or WC asthma were nearly twice those of patients with NTC or 'not' WC (NWC) asthma, respectively.

**Figure 3:**
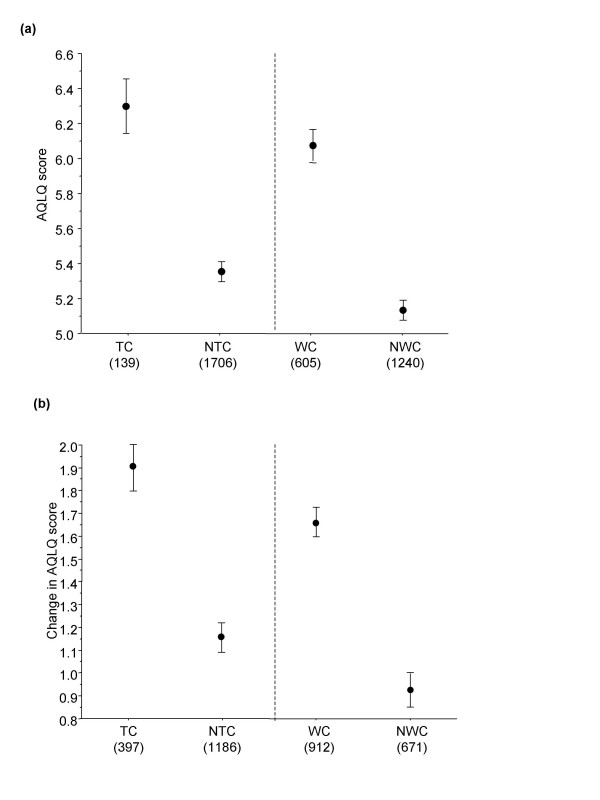
(a) Asthma Quality of Life Questionnaire (AQLQ) score by control status at Week 12 (mean and 95% confidence interval) and (b) Change in AQLQ score from baseline to Week 52 by control status at Week 52 (mean and 95% confidence interval). Patients with TC asthma has significantly higher AQLQ scores compared to those with NTC asthma, and WC patients had significantly higher AQLQ scores compared to those NWC. *Key: *TC: Patients with totally controlled asthma. NTC: Patients with well controlled or not well controlled asthma. WC: Patients with either well controlled or totally controlled asthma. NWC: Patients with not well controlled asthma. Figures in brackets are number of patients per group.

The asthma control measures also had good discriminative properties when compared with AQLQ scores at Week 12 (Figure 3a) and change in AQLQ scores from baseline to Week 52 (Figure 3b). Higher (better) AQLQ scores and greater improvement in scores were associated with achieving control status compared to not achieving control status, for both TC and WC asthma. The differences between the mean AQLQ scores at Week 12 and the mean change from baseline scores at Week 52 were statistically significant (p < 0.001) for both TC and WC asthma compared to NTC and NWC, respectively.

We further explored the relationship between asthma control status and percent predicted FEV_1 _by comparing percent predicted FEV_1 _for those patients achieving control both overall and for each of the individual asthma control components at Week 52, for both TC and WC asthma. Model estimates for overall control status and the individual criteria are shown for patients with TC vs NTC asthma, and WC vs NWC asthma in Table 3. The amount of variance (R^2^) in percent predicted FEV_1 _at Week 52 accounted for by overall control status was greater than that attributable to the individual control status components (TC = 6%, WC = 5%), with the exception of PEF where the higher R^2 ^value was predictably attributable to the strong relationship between these two measures of airflow limitation. Results suggest that the composite asthma control have measures better discriminative properties compared to the individual asthma control status components alone.

**Table 3:** Relationship between overall control status or control status criteria and percent predicted FEV_1_at Week 52

Control status	Model	Estimate (standard error)	p-value	R^2^
Totally controlled	Overall	94.36 (0.639)	< 0.0001	0.06
	Symptoms	90.22 (0.555)	< 0.0001	0.02
	N-T awake	88.04 (0.416)	< 0.0001	0.01
	β_2 _use	90.23 (0.514)	< 0.0001	0.03
	PEF	93.52 (0.429)	< 0.0001	0.17
	Exac.	87.18 (0.373)	0.02	0.00
	EV	87.10 (0.375)	0.4	0.00

Well controlled	Overall	90.41 (0.459)	< 0.0001	0.05
	Symptoms	88.58 (0.419)	< 0.0001	0.02
	N-T awake	88.04 (0.416)	< 0.0001	0.01
	β_2 _use	89.75 (0.446)	< 0.0001	0.04
	PEF	93.52 (0.429)	< 0.0001	0.17
	Exac.	87.18 (0.373)	0.02	0.00
	EV	87.10 (0.375)	0.4	0.00

Symptoms = Daytime symptoms; N-T awake = Night time awakening; β_2 _use = Use of rescue β_2_-agonist; PEF = Peak expiratory flow rate; Exac. = exacerbations; EV = Emergency visits; AE = Treatment-related adverse events.

To test the ability of the asthma control definitions to summarise other measures of disease activity, we examined the relationship between asthma control status and the two reference criterion: percent predicted FEV_1 _and AQLQ score. The model showed that both reference criteria were independently significantly related to asthma control status (p < 0.0001). The two criteria together accounted for 7% of the variance in TC asthma and 19% of the variance in WC asthma. This suggests that although these two measures of disease activity are summarised by both definitions of asthma control, the majority of the variance (TC = 93% and WC = 81%) is attributable to other unidentified factors.

### Predictive validity

To test the ability of asthma control to predict future markers of disease activity, we compared asthma control status at Week 12 to percent predicted FEV_1 _and the AQLQ score at Week 52 (Table 4). The models show that asthma control status has good predictive validity indicating a significant difference in mean scores at Week 52 for both TC and WC patients against NTC and NWC patients, respectively.

**Table 4:** Predicting Asthma Quality of Life Questionnaire (AQLQ) score and percent predicted forced expiratory volume in 1 second (FEV_1_) at Week 52 from control status at Week 12

		AQLQ score			Percent predicted FEV_1_		
		
		Mean	Difference	p-value	Mean	Difference	p-value
Totally controlled	Yes	6.48	0.63	< 0.0001	95.7	9.7	< 0.000
	No	5.84			86.0		1

Well controlled	Yes	6.34	0.67	< 0.0001	91.5	6.9	< 0.000
	No	5.67			84.6		1

## Discussion

The results of this study provide evidence that the composite measures of TC and WC, derived from international guidelines, are valid instruments for defining and measuring asthma control. Although recent guidelines [1-3] have suggested specific criteria for assessing asthma control there are a limited number of measures available for assessing comprehensive asthma control [14,34,35]. Individual criteria alone do not sufficiently encompass the full spectrum of the impact of the disease on patients and a more global approach to the definition of control was required [5]. There was a need for a simple to use, evidence-based, practical measure that indicated when a patient's asthma had achieved the target level/s of control suggested in clinical guidelines, and that might serve as a goal for treatment of patients in clinical practice.

We evaluated the psychometric properties of TC and WC definitions as measures of asthma control in several ways.

In order for each individual component to make a necessary and sufficient contribution to the TC and WC composite definitions of asthma control they should each be relatively independent of each other [26]. We showed that correlations among the individual components were low to moderate, supporting the independence of each element's contribution to the overall definition. This is commensurate with what we would expect from such measures, given that strong correlations would suggest that some of the criteria were providing redundant information and were therefore not necessary components of the composites. Traditional measures of construct validity, such as a Cronbach's alpha, were not calculable with this type of outcome measure. However, we believe that we have demonstrated that the TC and WC measures have construct validity with the tests we have performed.

In order for the measures of asthma control to have practical value in terms of clinical practice, it is essential that they have the ability to discriminate between patients with differing levels of asthma both cross-sectionally and longitudinally. One of the difficulties of assessing the validity of composite measures is the requirement for markers of disease activity that are not already elements of the composite as sources of comparison – also known as reference measures. We were able to evaluate the discriminative properties of the TC and WC measures against two established measures of disease activity. FEV_1 _is a measure of airflow limitation in airways disease and the AQLQ score is a measure of quality of life in asthma as measured by patients. These two indices may be considered representative of different elements of disease activity given the widely reported low correlations between FEV_1 _and quality of life measures [36-39] and the poor relationship between change in FEV_1 _and decline in overall health [40]. Using these two independent reference criteria we have demonstrated that the measures of TC and WC asthma have good cross-sectional and longitudinal discriminative properties when compared against other markers of disease activity.

We have further confirmed the discriminatory properties of the TC and WC measures by demonstrating that they have a greater ability to discriminate between levels of percent predicted FEV_1 _severity compared to the individual criteria alone. The ability of the control measures to encompass a range of markers of disease activity was shown by its independent relationship to both FEV_1 _and AQLQ scores. Moreover, both of these reference criteria combined accounted for less than 10% of the variability in asthma control, suggesting that the TC and WC measures encompass more than just these two elements and in fact reflect a more global definition of asthma control.

For the purpose of managing asthma in the individual patient and for evaluating new treatments in clinical trials, it is preferable for any new measure to have predictive validity. In other words, that it has the ability to reliably predict future changes in disease activity. We have shown that the measures of TC and WC asthma 12 weeks into the GOAL study differentiate between FEV_1 _and AQLQ scores 1 year later and hence that they have good predictive validity, at least within the treatment conditions of the GOAL study, where treatment was increased and then maintained for the duration of the study.

A limitation of the current validation of these control measures is that it is restricted by the design and parameters recorded in the GOAL study on which it is based. Diary card recall was limited to 1 week and the study recruited patients with poorly controlled asthma. The validity of the measures over longer recall periods with different populations would have to be re-established; in the event that the guidelines are updated in accordance with advances in research, the control measures would similarly need to be adjusted and revalidated.

The control measures evaluated in this study include patient-reported subjective endpoints (e.g. symptom score) and a patient-reported measure of lung function. They do not, however, include markers of asthma pathophysiology or measures of lung function taken in the clinic. Asthma is a complex entity comprising a range of factors and although it is important to evaluate the physiological features of asthma, it is equally important to take into account subjective measures, as these influence service use, effective self-management and compliance. Our understanding of asthma and our ability to control it are enhanced by both perspectives.

We have demonstrated that this instrument has good psychometric properties and that it is effective as a tool designed to aid clinical management. These measures of asthma control were designed to be consistent with GINA guidelines and therefore the component endpoints were not selected according to the principles of classical test theory, e.g. by using principal components analysis [24-27]. Similarly, because the main aim of the measures was to serve as an index of achieved control, a graded scoring system was not deemed a necessary function. It is acknowledged, however, that for other clinical uses, such as the evaluation of disease progression or response to a new treatment, there are advantages in using a quantifiable rather than categorical outcome measure. Categorical outcome measures are less useful for clinical purposes as they are not able to detect subtle changes in the components of asthma (for example, a small improvement or deterioration in PEF or symptoms), particularly in patients that do not achieve the threshold level of control (TC or WC).

The Asthma Control Test [15], the Asthma Control Questionnaire (ACQ) [35] and Asthma Control Scoring System [20] are examples of measures that include composites of endpoints and provide numeric scores indicating asthma control status. Each has undergone a measure of validation in different clinical and research settings and are offered as an alternative to the GINA categorical measure of control in the latest version of the GINA guidelines. However, unlike TC and WC, none is based directly on, nor has sought to validate, the endpoints and goals contained in the guidelines, which was the purpose of the current study.

A limitation of many composite measures, including those evaluated in the present study, is the fact that each component endpoint assumes equal importance in either the categorisation or contribution to the overall score. The weighting of the component endpoints in composite measures is an issue that requires further research: there is a need for a measure of asthma control that is guideline-based, sensitive, valid and reliable; that includes validated cut-points for major control milestones; and reflects the relative importance of component parameters.

## Conclusion

In conclusion, this validation study has shown that the psychometric properties of the asthma control measures of TC and WC asthma, as used in the GOAL study, are consistent with an instrument that has good reliability, discriminative ability and predictive validity. These control measures are valid functional indicators of clinical status that can be used in the evaluation of the efficacy of asthma treatments and the overall management of patients with asthma.

## Competing interests

SS received a consultancy remuneration from GlaxoSmithKline for her contribution to the research paper.

## Authors' contributions

KB and BM performed the statistical analyses. SS and EB provided intellectual input, writing and review of the data and paper. All authors read and approved the final manuscript.

## References

[B1] Global Initiative for Asthma (2002). Global Strategy for Asthma Management and Prevention: NHLBI/WHO workshop report.

[B2] British Thoracic Society and Scottish Intercollegiate Guidelines Network (2003). British guideline on the management of asthma. Thorax.

[B3] National Asthma Education and Prevention Program (1997). Guidelines for the Diagnosis and Management of Asthma Expert Panel Report 2.

[B4] Bateman ED, Bousquet J, Braunstein GL (2001). Is overall asthma control being achieved? A hypothesis-generating study. Eur Respir J.

[B5] Bateman ED, Frith LF, Braunstein GL (2002). Achieving guideline-based asthma control: does the patient benefit?. Eur Respir J.

[B6] Celli BR, Cote CG, Marin JM, Casanova C, Montes de Oca M, Mendez RA, Pinto Plata V, Cabral HJ (2004). The body-mass index, airflow obstruction, dyspnea, and exercise capacity index in chronic obstructive pulmonary disease. N Engl J Med.

[B7] British Thoracic Society (1997). The British guidelines on asthma management 1995 review and position statement. Thorax.

[B8] Global Initiative for Asthma (1998). Global Strategy for Asthma Management and Prevention NHLBI/WHO Workshop Report March 1993 Revised November 1998.

[B9] Quanjer P, Lebowitz M, Gregg I, Miller M, Pedersen O (1997). Peak expiratory flow: conclusions and recommendations of a Working Party of the European Respiratory Society. Eur Respir J.

[B10] Jones K, Mullee M, Middleton M, Chapman E, Holgate S (1995). Peak flow based asthma self-management: a randomised controlled study in general practice. Thorax.

[B11] Gibson P, Wong B, Hepperle M, Kline P, Girgis-Gabardo A, Guyatt G (1992). A research method to induce and examine a mild exacerbation of asthma by withdrawal of inhaled corticosteroid. Clin Exp Allergy.

[B12] Fabbri L, Beghe B, Caramori G, Papi A, Saetta M (1998). Similarities and discrepancies between exacerbations of asthma and chronic obstructive pulmonary disease. Thorax.

[B13] Silkoff P, Martin R (1998). Pathophysiology of nocturnal asthma. Ann Allergy Asthma Immunol.

[B14] Juniper EF, O'Byrne PM, Guyatt GH, Ferrie PJ, King DR (1999). Development and validation of a questionnaire to measure asthma control. Eur Respir J.

[B15] Nathan RA, Sorkness CA, Kosinski M, Schatz M, Li JT, Marcus P, Murray JJ, Pendergraft TB (2004). Development of the asthma control test: a survey for assessing asthma control. J Allergy Clin Immunol.

[B16] Kendrick A, Higgs C, Whitfield M, Laszlo G (1993). Accuracy of perception of severity of asthma: patients treated in general practice. BMJ.

[B17] Nowak R, Pensler M, Sarkar D, Anderson J, Kvale P, Ortiz A (1982). Comparison of peak expiratory flow and FEV1 admission criteria for acute bronchial asthma. Ann Emerg Med.

[B18] Sawyer G, Miles J, Lewis S, Fitzharris P, Pearce N, Beasley R (1998). Classification of asthma severity: should the international guidelines be changed?. Clin Exp Allergy.

[B19] Bateman ED, Boushey HA, Bousquet J, Busse WW, Clark TJH, Pauwels RA, Pedersen SE (2004). Can guideline-defined asthma control be achieved? The Gaining Optimal Asthma ControL Study. Am J Respir Crit Care Med.

[B20] Boulet L-P, Boulet V, Milot J (2002). How should we quantify asthma control? A proposal. Chest.

[B21] Jones PW (1992). Measurement of health in asthma and chronic obstructive airways disease. Pharmaceutical Medicine.

[B22] Guyatt GH, Walter S, Norman G (1987). Measuring change over time: assessing the usefulness of evaluative instruments. J Chron Dis.

[B23] Jones PW, Quirk FH, Baveystock CM (1991). The St George's Respiratory Questionnaire. Respir Med.

[B24] Juniper EF, Buist AS, Cox FM, Ferrie PJ, King DR (1999). Validation of a standardized version of the asthma quality of life questionnaire. Chest.

[B25] Jones PW (1998). Testing health status ("quality of life") questionnaires for asthma and COPD. Eur Respir J.

[B26] Nunnally JC, Bernstein IH (1994). Psychometric Theory.

[B27] Fayers P, Hays R (2005). Assessing Quality of Life in Clinical Trials.

[B28] Juniper EF, Guyatt GH, Epstein RS, Griffith LE (1992). Evaluation of impairment of health-related quality of life in asthma: development of a questionnaire for use in clinical trials. Thorax.

[B29] Rowe B, Oxman A (1993). Performance of an asthma quality of life questionnaire in an outpatient setting. Am Rev Respir Dis.

[B30] Juniper EF, Johnston PR, Borkhoff CM, Guyatt GH, Boulet LP, Haukioja A (1995). Quality of life in asthma clinical trials: comparison of salmeterol and salbutamol. Am J Respir Crit Care Med.

[B31] Leidy N, Coughlin C (1998). Psychometric performance of the Asthma Quality of Life Questionnaire in a US sample. Qual Life Res.

[B32] Juniper EF, Guyatt GH, Ferrie PJ, Griffith LE (1993). Measuring quality of life in asthma. Am Rev Respir Dis.

[B33] Juniper EF, Guyatt GH, Willan A, Griffith LE (1994). Determining a minimal important change in a disease-specific quality of life questionnaire. J Clin Epidemiol.

[B34] Katz PP, Yelin EH, Smith S, Blanc PD (1997). Perceived control of asthma: development and validation of a questionnaire. Am J Resp Crit Care Med.

[B35] Juniper EF, O'Byrne PM, Ferrie PJ, King DR, Roberts JN (2000). Measuring asthma control. Clinic questionnaire or daily diary?. Am J Respir Crit Care Med.

[B36] Mahler DA, Tomlinson D, Olmstead EM, Tosteson ANA, O'Connor GT (1995). Changes in dyspnea, health status, and lung function in chronic airways disease. Am J Respir Crit Care Med.

[B37] Ketelaars CAJ, Schlosser MAG, Mostert R, Huyer Abu-Saad H, Halfens RJG, Wouters EFM (1996). Determinants of health-related quality of life in patients with chronic obstructive pulmonary disease. Thorax.

[B38] Jones PJ (1994). Quality of life, symptoms and pulmonary function in asthma: long-term treatment with nedocromil sodium examined in a controlled multicentre trial. Eur Respir J.

[B39] Engstrom C-P, Persson L-O, Larsson S, Ryden A, Sullivan M (1996). Functional status and well being in chronic obstructive pulmonary disease with regard to clinical parameters and smoking: a descriptive and comparative study. Thorax.

[B40] Spencer S, Calverley PMA, Burge PS, Jones PW (2001). Health status deterioration in patients with COPD. Am J Resp Crit Care Med.

